# Identification of the Domain Structure Defects of a Radially Magnetized Rubber–Ferritic Conglomerate

**DOI:** 10.3390/ma16093487

**Published:** 2023-04-30

**Authors:** Karolina Popowska, Szymon Gontarz, Przemysław Szulim

**Affiliations:** Institute of Vehicles and Construction Machinery, Warsaw University of Technology, Narbutta 84, 02-524 Warsaw, Poland

**Keywords:** diagnostic parameters, magnetic-based diagnostics, magnetic encoder ring, magnetic domain structure

## Abstract

Modern solutions in materials engineering are designed not just for the improvement in the mechanical or electromagnetic properties of materials but also to begin to fulfill specific functional roles. A good example of such a modern solution is a composite made of steel and rubber–ferritic conglomerate, which is the research object of the article. The composite, when properly magnetized, can act as a magnetic encoder ring for reading the angular displacement, speed, or acceleration parameter. The paper addresses the problem of identifying and assessing the defects of the magnetic encoder ring domain structure in the form of a radially magnetized ring. It discusses the essential types of the ring’s degradation, such as mechanical, thermal, and magnetic, and presents problems related to the identification of emerging defects. The conducted research allows a better understanding of the degradation process in the context of magnetic encoder ring reliability. Based on the conducted research on the proposed test stand, it is possible to track the progressive degradation related to each effect. These degradation case analyses consider both quantitative and qualitative changes in the encoder ring’s domain structure. The proposed parameters show the possibilities and perspectives for detecting the ring’s defects in the early stage of its development. Solely such an approach will allow for proper exploitation and extension of the applicability of this kind of ‘intelligent material’. Additionally, the developed parameters for the encoder ring’s defects detection can support the progress of rapidly evolving methods for diagnosing mechanical systems based on a signal from such an element.

## 1. Introduction

**Figure 1:**
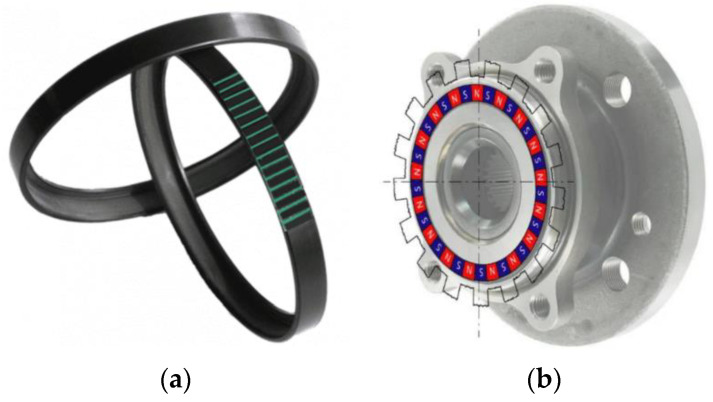
Selected types of magnetic encoder rings: (**a**) Circumferentially magnetized encoder ring and (**b**) radially magnetized encoder ring [8,9].

Modern technology development almost excludes the existence of technical objects developed within one engineering discipline. At present, these are complex objects that can generally be called mechatronics. Interferences and permeation of various disciplines cause objects to acquire new properties and functionalities. This direction is being pursued in parallel with a clear tendency toward the miniaturization of technical solutions. When considering modern machines whose primary task is performing a rotational movement, it is clear that systems for measuring the parameters of this motion have become necessary. In the beginning, they were independent, with various measurement paths, such as RLC bridges, strobotron circuits, and tachometric generators [1,2,3], and later more integrated devices, such as in the form of discs rotating with a shaft and inductive sensors [4]. The latest solutions use magnetic encoder rings cooperating with appropriately selected magnetometers [5]. Considering the integration and miniaturization mentioned above [5,6], steel and rubber–ferritic conglomerate composites were created. After appropriate magnetization, it can act as a magnetic encoder for reading parameters, such as angular position, velocity, or angular acceleration [7]. Moreover, such an element may be a rolling bearing sealing (Figure 1b), which means that only minimal interference in the object’s structure is enough to obtain information about the rotational movement.

In science and industry, magnetic encoders are applied to various topics. The information about rotational speed or angular displacement obtained through them is used for real-time machine monitoring, correcting the operation of machines, and controlling regulation systems, or, for example, ensuring the continuity and maintenance of the required parameters of production processes [10,11]. All these functions will be fulfilled provided that the magnetic encoder ring and magnetometer [12,13] are well maintained. Recently, it has been indicated that magnetic encoder rings can also act as a source of diagnostic information by providing a signal describing the unevenness of the rotary motion correlated with the technical condition of the rotary machine [5]. In this case, the quality and reliability of the signal from the encoder ring are even more critical, which, as a specific material engineering product, is subjected to many specific influences that may cause its degradation. Notably, imperfections not only result in the production process but also may arise during operation. Then, the range of factors influencing the encoder is much wider. Therefore, the behavior of the domain structure is diverse.

We can see from this that the magnetic encoder is the key element for the correct operation of the entire system. Assuming that the relevant standard guidelines are met [10], such as assembly criteria and characteristic parameters of the system elements, it is the encoder quality that can lead to a situation where the obtained information is erroneous. For encoders in automotive applications, standard suppliers [10,11,12] do not clearly indicate the parameters for assessing the quality of the magnetic field distribution and the amplitudes of the magnetic induction domains. Additionally, with regard to quality assessment requirements, suppliers are not completely consistent with each other. As a result of the analysis of that standard, three measures can be indicated: the number of domain pairs Ndp; their dimensions (e.g., Dw—domain width); and the minimum limit value of domain amplitudes measured from a certain distance $\left|B_{min}\right|$. These parameters have their reference values specified in the standards. There are some gaps in terms of measurement and diagnostic parameters. For example, it seems advisable to consider the width of the domains measured on a circumference with a nominal radius R to read the cumulative error of the width of the domains or to analyze the waveform of the magnetic induction value measured on that circumference.

Meeting the parameters for assessing the quality of encoders is necessary to obtain a reliable and repeatable measurement of rotational speed. Therefore, meeting this parameter type in more advanced applications, such as measuring the speed and angular position in devices such as wind turbines or fast and precise industrial robots, will be all the more important. One should also consider the signal from the encoder as a diagnostic signal of the rotating machine, the diagnosis of which will not be reliable without certainty as to the encoder–magnetometer measuring system. Understanding the nature of degradation, together with the possibility of their identification, can be an important contribution wherever encoder rings are used as a source of information about rotational motion parameters. Therefore, the aim of this article is to both identify and assess the defects of a magnetic encoder ring domain structure. Section 2 describes a proposal for a universal measurement stand and experiment conducted with the use of that stand. The next chapter presents the results of the experiments. The results include the use of the proposed diagnostic parameters, the task of which is the reliable identification of the defects at an early stage of their development. The last chapter discusses the possibility of presenting diagnostically oriented analysis of magnetic encoder rings.

## 2. Measurement Method

Magnetic encoder defects can be broadly categorized as those caused by manufacturing or operation. To make the topic more specific, the paper refers to the faults that could occur during operation. Magnetic encoders work with elements and mechanical systems. Due to a malfunction on the mechanical side, the encoder can be subjected to abrasion. The ring, in addition to the aforementioned function of providing information on the rotational speed of the shaft, also acts as a seal for the hub bearing in the machine. Due to insufficient grease in the bearing, the mating elements may wear and overheat. When we consider the magnetic encoder in a vehicle’s ABS system, degradation may occur as a result of an increase in temperature in the drive system. Rapid braking or braking in the event of a high load on the drive system (driving down a steep slope) results in an increase in temperature to above 673 K. It is also worth mentioning that the number of electric cars is currently increasing. In the drive system of such a vehicle, in the event of engine damage (both mechanical and electric), the phenomenon of magnetic flux leakage may occur, which may lead to the demagnetization of the encoder domain structure [14].

**Figure 2:**
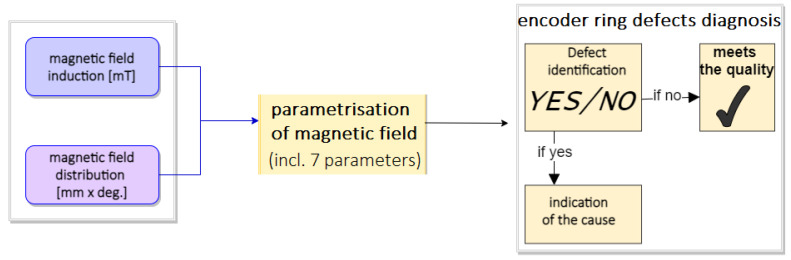
The novel method measurement procedure.

An appropriate measurement station and methodology are proposed to detect the defects described in this chapter. The proprietary stand, in conjunction with the parametric evaluation, became the basis for a novel measurement method used in this research. The schematic diagram is shown in Figure 2.

### 2.1. Hardware and Software Architecture of the Stand

**Figure 3:**
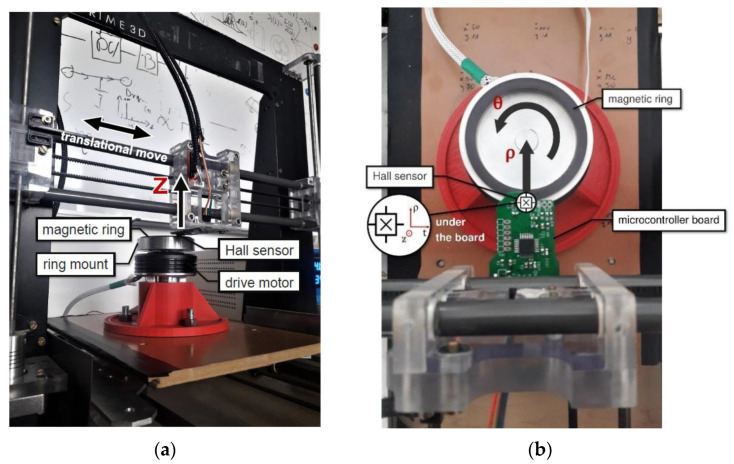
Measurement stand: (**a**) front view and (**b**) top view.

The measuring device had the form of a 4-axis scanner, with the possibility of making 3 translational movements (X, Y, Z) and one rotary movement. During the scanning of the research object, the stand combines rotary and translational motion. Due to such a combination of movements, the distribution of the magnetic field of the encoder is determined. The advantage of introducing a rotational motion in the encoder measurement is the maintenance of a polar coordinate system reflecting the natural working conditions of the target encoder and sensor system. The test stand had the ability to accurately position the sensor on the vertical axis (Figure 3a) to maintain the same distance from the encoder surface throughout the entire measurement. The scanner resolution for the range of translational movements was at the level of 0.1 mm, while for the rotational movement, it was at 0.023 degrees. The measurement of the displacement on the X, Y, and Z axes was based on counting the impulses of the stepper motors (with zeroing after reaching the extreme position detected by optical limit sensors) only, while the axis of rotation was equipped with an additional absolute magnetic encoder with a resolution of 14 bits. Figure 3 shows the measuring stand with the tested object.

As a measuring element, a Hall magnetometer was used in the stand (Figure 3b) [15]. The measuring axis (X-axis) of the sensor was tangent to the curvature of the magnetic encoder, and the Y-axis was radial and followed the trajectory of the head—ρ (Figure 3b), while the Z-axis was directed vertically upward (Figure 3a).

By keeping the distance constant throughout the measurement, it is possible to obtain a repeatable measurement result of the magnetic field. In the case of measuring encoders, the use of a precise angular measurement significantly increases the result accuracy because the parameters of the number of domain pairs Ndp, domain widths Dw, and the minimum limit value of domain amplitudes $\left|B_{min}\right|$ are determined in the circumferential direction. Measurements carried out in this way provide signals that have been subjected to a detailed diagnostically oriented analysis.

### 2.2. Experiment

**Figure 4:**
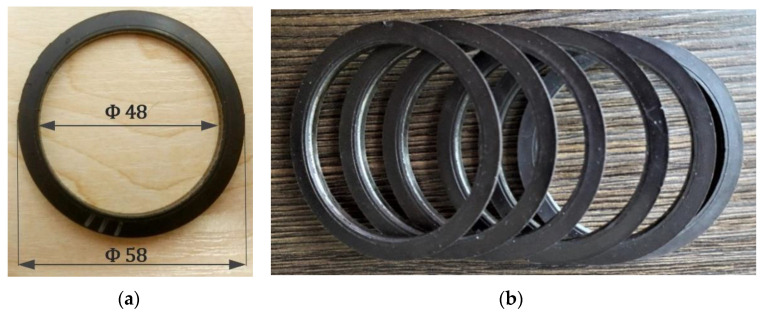
Object of the experiment. i.e., the magnetic encoder ring: (**a**) encoder with marked characteristic dimensions—internal and external diameter and (**b**) copies of encoder rings are marked for testing purposes.

Based on the described measuring station, experiments were planned in which magnetic encoders were subjected to various types of degradation. Figure 4 shows the object of the experiment—a magnetic encoder. The encoder ring samples were obtained as a courtesy by the encoder ring’s manufacturer. It is a standard magnetic ring, which is one of the seals of the rolling bearing. Such constructions are currently used, for example, in the automotive industry, and are a key element in ABS/ASR systems to determine efficiency.

The nominal magnetic properties and geometric dimensions of the shown encoder ring (Figure 4), according to the standard [10], are listed as follows:

- Number of domain pairs Ndp=48;
- Inner diameter Di=48 mm and outer diameter Do=58 mm (Figure 4a);
- Diameter of the nominal reading circumference of the measurements $D_{mean}=53\;\mathrm{mm}$; hence, the width of a single domain Dw=1.64 mm;
- The minimum, limiting value of domain magnetic induction amplitudes $\left|B_{min}\right|$ dependent on the measurement distance [10].

For the purposes of the experiment, the degradation of magnetic encoders was reproduced in laboratory conditions. Subsequent magnetic encoders were subjected to the following impacts: 1—temperature; 2—magnetic field; and 3—mechanical abrasion. The controlled increase in the intensity of these interactions resulted in successive degrees of degradation of the magnetic information contained in the rings. The table below (Table 1) shows the degree of the severity caused by various types of damage to the encoders.

The proposed experiment has a useful purpose because thermal, mechanical, or magnetic degradation may occur during the stage of operation or even production.

## 3. Measurement Results

Each measurement of the 15 rings was made on the proprietary measuring stand (Section 2.1), taking into account 55 waveforms of the induction value recorded on successive encoder circuits with given radii. This measurement method makes it possible to obtain the distribution of the magnetic field over the encoder surface. The measurements were carried out at a fixed distance of 0.3 mm from the magnetometer’s sensitive element to the encoder surface. A constant distance between the sensor and the encoder surface throughout the measurement allows the control of the distance from the signal source (encoder ring’s surface) to the measured magnetic field induction value. The above conditions ensure reliable magnetic induction measurements, which decrease with increasing distance from the signal source [13].

### 3.1. Mechanical Degradation

Five magnetic encoder ring samples were subjected to five different mechanical degradation intensities. The rubber–ferritic layer of the encoder ring, with a nominal thickness of 0.70 mm, was abraded from a depth of 0.23 mm to 0.50 mm (Table 1). In addition, specific real operating conditions were simulated, i.e., tearing of the magnetic layer, which may occur during the mechanical abrasion process (sample “0.30 + cavities”). The encoder rings were abraded perpendicular to their surface due to mechanical friction.

Preliminary research has shown that mechanical degradation can be observed in the change in the magnetic field of the damaged encoder ring that is directly proportional to the abrasion depth. Additionally, there is a decrease in the domain amplitudes and a flattening of the distribution of magnetic induction values on the encoder ring radial waveform.

**Figure 5:**
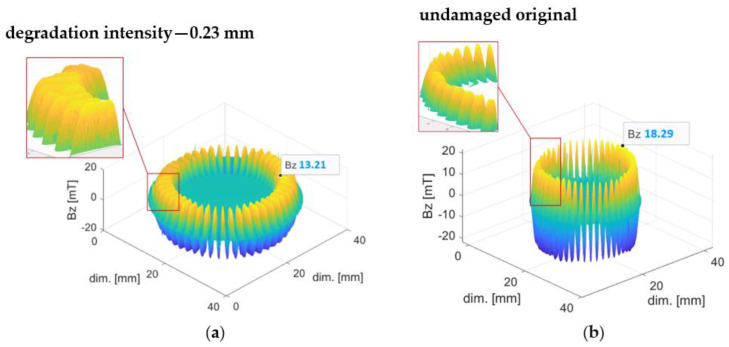
3D magnetic induction distributions of the mechanically degraded encoder ring for an abrasion depth of 0.23 mm (**a**) and its undamaged prototype (**b**).

Figure 5 shows 3D visualizations of magnetic field induction distributions of the encoder ring subjected to regular mechanical degradation (Figure 5a) and its undamaged prototype (Figure 5b). The data labels show the magnetic induction peak values of the encoder ring domains. The magnetic induction Bz peaks of the mechanically abraded ring domains had a flattened shape (Figure 5a), while the domain peaks of the undamaged encoder ring had a slender shape (Figure 5b), which can be observed in the domain magnifications on the graphs.

**Figure 6:**
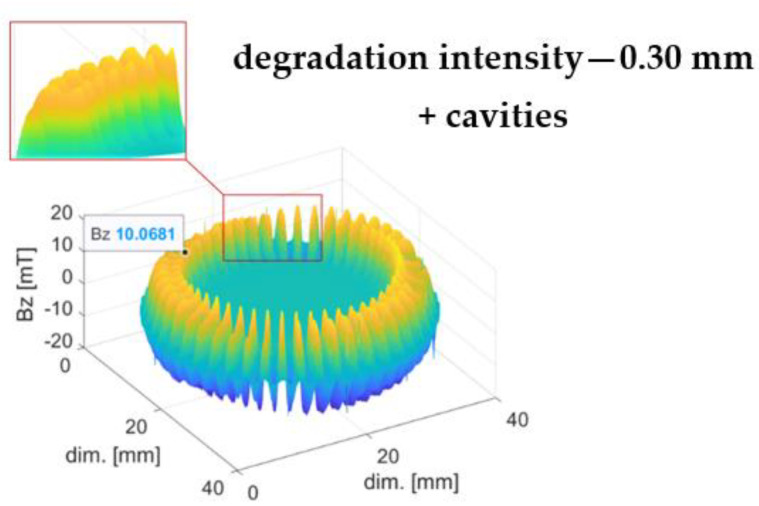
3D magnetic induction distribution of mechanically degraded material cavities. The material was abraded to a depth of 0.30 mm.

Figure 6 shows the magnetic field induction distribution of a ring subjected to an irregular, specific type of mechanical degradation with material cavities. The material was abraded to a depth of 0.30 mm. On the close-up view of the graph area, the shapes of the sample’s domain magnetic induction peaks are visible.

The magnetic induction Bz values of the encoder rings subjected to mechanical degradation were directly related to the degradation depth d (both in the regular case—Figure 5b and in the specific one, i.e., mechanical abrasion with material cavities—Figure 6). The increased mechanical degradation depth d of the rubber–ferritic layer caused a decrease in the magnetic induction value Bz of the encoder ring domains (1).

(1) $$\;\;\;\;\;\frac{d_{n}}{Bz_{n}}=\frac{d_{0}}{Bz_{0}},\;for\;d_{n}>d_{0}=0\;\mathrm{mm},\;\;Bz_{n}>Bz_{0}$$

where $d_{n}$ is the degradation depth of the *n*-th probe, $d_{0}$ is the initial degradation depth of the undamaged sample, $Bz_{n}$ are the peak values of the magnetic induction domains of the *n*-th sample, and $Bz_{0}$ is the magnetic induction peak values of the undamaged sample’s domains.

### 3.2. Thermal Degradation

**Figure 7:**
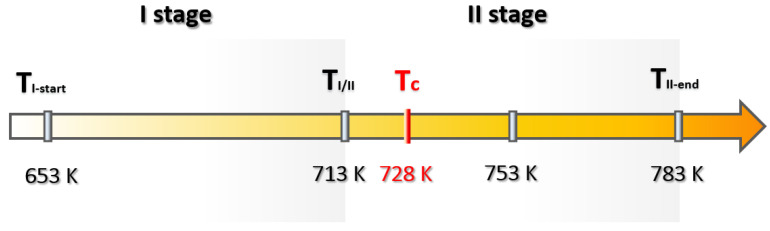
Schematic diagram of the thermal degradation process.

Another five magnetic encoders were subjected to five different degrees of temperature exposure ranging from 293 K (ambient temperature) to 793 K (Table 1). In the thermal degradation process, stage II damage can be distinguished for the two temperature ranges. Stage I included temperatures from 653 K to 713 K (for 653 K and 713 K measurements), and stage II ranged from 713 K to 793 K (for 753 K and 783 K measurements, respectively), as shown in Figure 7. The Curie temperature for the ferritic powder used in the encoder ring was 728 K [16] (Figure 7).

**Figure 8:**
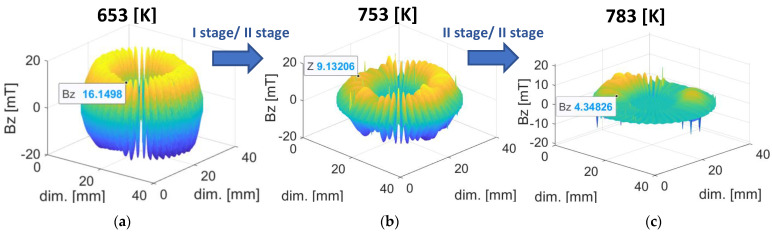
Thermal degradation stages: (**a**) 653K temperature, (**b**) 753K temperature, (**c**) 783K temperature.

Figure 8 shows 3D visualizations of the magnetic field of the encoder ring for both stage I and stage II of thermal degradation.

In stage I of thermal degradation, the decrease in the magnetic field induction value of the ring domains was slight, on the order of a few mT (Figure 8a). Such a reduction in the magnetic induction value of the encoder ring did not exceed the required magnetic induction limit values contained in the standards [10] (Section 1). Therefore, the magnetic ring did not lose its encoder functionality. At stage II of thermal degradation, for the observed temperature of 743–763 K (Figure 8b), there was an abrupt decrease in the magnetic induction value in comparison with degradation stage I, and the ring’s domain structure was reorganized, which did not occur in stage I. It can be concluded that the abrupt degradation of the magnetic induction occurred before the observed temperature of 743 K, after exceeding the Curie temperature (728 K) for the ferritic powder used in the conglomerate [16]. On a certain part of the circumference, the rubber–ferritic conglomerate completely lost its magnetic properties, becoming paramagnetic, and the encoder domains were demagnetized. The composite of steel and rubber–ferritic conglomerate lost the encoder functionality.

The reason for circumferentially nonuniform thermal degradation (Figure 8c) was the nonuniform distribution of heat during the experiment caused by the natural diffusion of gases into the environment, resulting in an unequal temperature intensity along the entire ring’s circumference length.

### 3.3. Magnetic Degradation

Another five encoders were magnetically degraded with five different degrees of damage. In the process of magnetic degradation, stage II could be distinguished due to the values of the intensity of the demagnetizing field. In stage I, the values of the destructive field ranged from 45 mT to 100 mT, and in stage II, the values were 120 mT and 145 mT.

**Figure 9:**
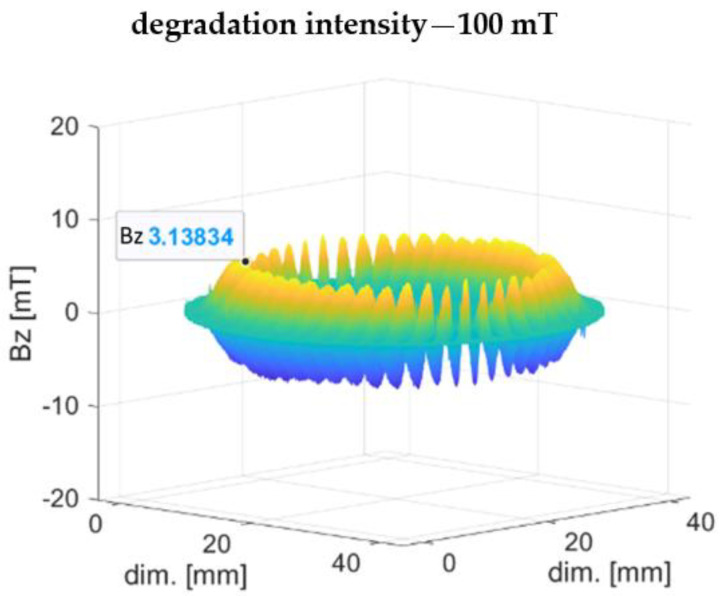
Magnetic field induction distribution of a ring subjected to 100 mT intensity of magnetic degradation.

Figure 9 shows a visualization of the encoder ring’s 3D magnetic induction distribution for stage I of magnetic degradation with a 100 mT intensity. For stage I of magnetic degradation (Figure 9 the magnetic induction peaks of the domains did not change their shape in comparison with the undamaged prototype (Figure 5b), unlike in the mechanical degradation case (Figure 5a), where the peak shape flattened. For stage I of magnetic degradation (Figure 9), domain magnetic induction decreased evenly along the radial direction, and the domain peak shape was slender, similar to the undamaged sample (Figure 5b).

Stage I of magnetic degradation was characterized by a decrease in the magnetic induction Bz peak values of the domains (Figure 9) relative to the magnetic induction values of the undamaged encoder ring (Figure 5b). The Bz values decreased with an increase in the intensity of the destructive magnetic field H(2).

(2) $$\frac{H_{n}}{Bz_{n}}=\frac{H_{0}}{Bz_{0}},\;for\;H_{n}>H_{0}=0\;\mathrm{mT},\;\;Bz_{n}>Bz_{0}$$

where $H_{n}$ is the intensity of the degrading magnetic field, $H_{0}$ is the initial degrading magnetic field intensity, $Bz_{n}$ is the peak value of the domain’s magnetic induction of the *n*-th sample, and $Bz_{0}$ is the peak value of the domain’s magnetic induction of the undamaged sample.

**Figure 10:**
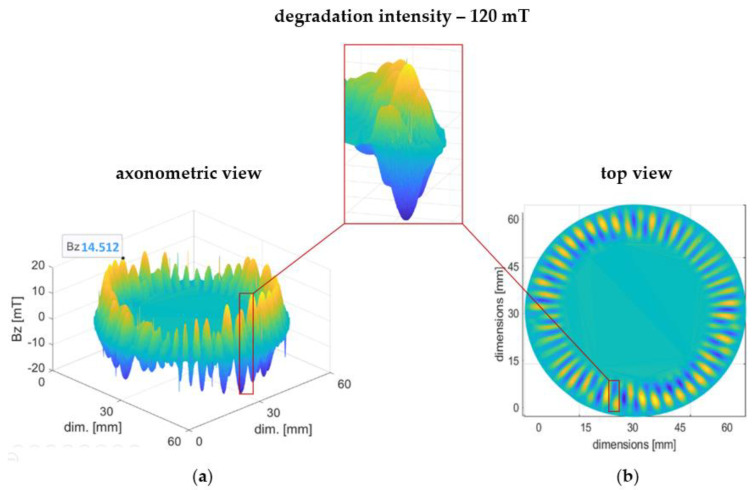
3D magnetic induction distribution of the ring subjected to magnetic degradation with an intensity of 120 mT. The axonometric view of the magnetic field distribution (**a**) and a top view (**b**). The red frame shows one of the domains in which magnetic induction changes its sign.

Figure 10 shows a visualization of the 3D magnetic induction distribution of the encoder ring in stage II of magnetic degradation. The red frame shows one of the domains, which in the radial direction changes its sign.

The ring (Figure 10) was subjected to one of the two highest intensities of degradation (Table 1) with an intensity of 120 mT.

In stage II of magnetic degradation, in comparison to stage I, there is an irregular decrease in the magnetic induction of the encoder ring circumference Bz (2) (Figure 10a). Moreover, there is a change in the ring’s magnetic field distribution; that is, the regular, radially magnetized domain structure becomes reorganized (Figure 10b). This causes sign changes in the domains in the encoder ring’s radial direction, which is manifested in the magnetic field distribution’s top view (Figure 10b) as the intermingling of blue and orange fields.

Using the capabilities of the measuring stand, a measurement and related parameters of the measured signal were proposed, which are further analyzed in the Section 4.

## 4. Parametric Analysis of the Results

A properly performed measurement with the use of the proposed stand allows for an initial assessment of the magnetic encoder. However, to obtain more detailed information on possible degradation and to give the verification functional characteristics, an appropriate parametric description of the tested object should be carried out. The vertical axis $B_{Z}$ was the direction of the magnetic field induction vector of the encoder domains considered in the analyses.

A similar approach can be found in the existing standards that can be used as measures to assess the quality of magnetic encoders. We found such parameters as the number of domain pairs Ndp, the width of domains Dw, and the minimum, limiting value of domain amplitudes $\left|B_{min}\right|$, which can be part of a parametric description. The standard provides only the values of the parameters without showing how they can be obtained; therefore, points 1 to 3 describe the method of extracting the mentioned parameters, obtained as a result of digital processing of data on the distribution of the magnetic field on the surface of the encoder and its magnetic induction values.

1. $\left|B_{min}\right|$ is the minimum absolute peak value of the magnetic induction of the encoder ring domains, Equation (3). $\left|B_{min}\right|$ is determined as a result of the analysis of peak values of magnetic induction of domains N and S (both positive $Bpeak^{+}$ and negative $Bpeak^{-}$) on the circumferential waveform of the magnetic induction signal of the encoder ring on the circumference with a given radius R. $\left|B_{min}\right|$ should be greater than or equal to the minimum absolute value of magnetic induction $\left|B_{\min\left(norm\right)}\right|$ specified in the standards [10,11], Equation (4).
  (3) $$\left|B_{min}\right|=MIN{\left(Bpeak^{+},\;Bpeak^{-}\right)}_{n}\;\left[\mathrm{mT}\right]$$
  (4) $$\left|B_{min}\right|\geq \;\left|B_{\min\left(norm\right)}\right|$$
  where $\left|B_{min}\right|$ is the measured minimum, absolute peak value of the magnetic induction value of the domains, $Bpeak^{+}\;\mathrm{and}\;Bpeak^{-}$ represent the positive and negative domain peaks, and $\left|B_{\min\left(norm\right)}\right|$ is the minimum absolute limit value of magnetic induction, specified according to the standards [10,11].
2. Ndp is the number of pairs of unlike domains, namely, N and S. The determination of the number of domain pairs Ndp is based on the number of single sine periods within the circumferential waveform of domain magnetic induction values. A single sine period denotes a set consisting of the positive domain peak $Bpeak_{}^{+}$ and negative domain $Bpeak_{}^{-}$.
  (5) $$Ndp=\overset{\frac{n}{2}}{\underset{i=1}{\cup }}{\left(Bpeak^{+},\;Bpeak^{-}\right)}_{n}\;\;$$
  where ∪  is the sum of sets symbol, $Bpeak_{}^{+}$ is the positive domain peak, and $Bpeak^{-}$ is the negative domain peak.
3. Dw is the width of a single domain, determined by the circumference with a given radius R. The measure of the domain width Dw is the number of consecutive measurement points whose induction values are the same sign: “+” or “−” (do not cross zero). To determine the number of positive/negative points in the magnetic induction vector B, the signum function was used, Equation (6):
  (6) sgn(B)=[1111111111−1−1−1−1−1−1−1…1111111]
4. Hence, the frequencies $f_{W\left(+\right)}$ and $f_{W\left(-\right)}$ of the “1” and “−1” characters, respectively, in the vector sgn(B) correspond to the widths of domains Dw with positive “+” and negative “−” signs.

(7) $$sgn\left(B\right)=\left[f_{W\left(+\right)}\;\;f_{W\left(-\right)}\;\ldots \;f_{W\left(+\right)}]=[10\;7\ldots 8\;\right]$$

(8) $$w=\frac{f_{W}}{\left|\vec{W}\right|}2\pi R\;\left[\mathrm{mm}\right]$$

**Figure 11:**
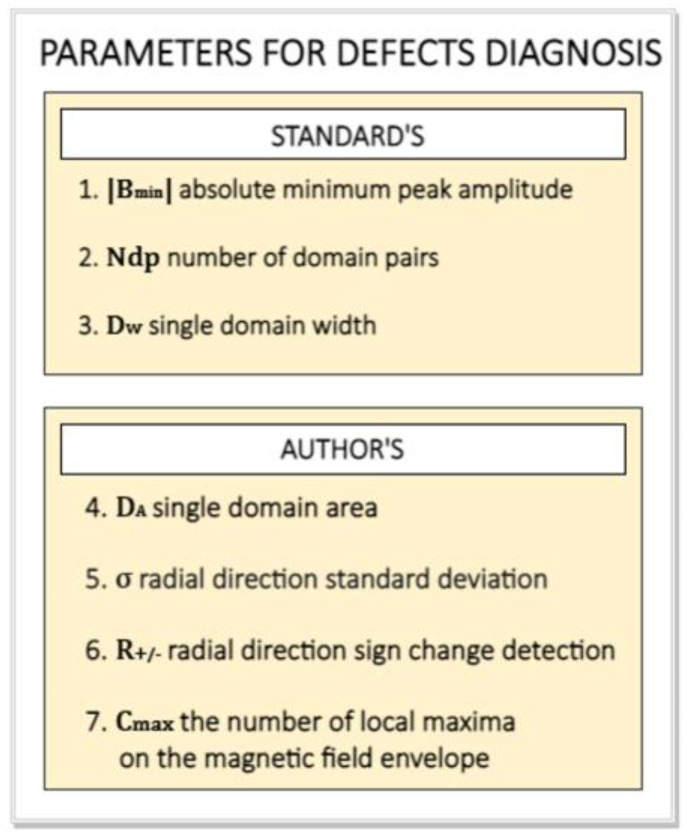
Parameters for magnetic encoder ring defect diagnostics in the scheme divided into standard parameters and those proposed by the authors of the article.

The set of parameters included in the standards [10,11], i.e., 1—$\left|B_{min}\right|$, 2—Ndp and 3—Dw—is not sufficient for unambiguous identification and determining the causes of specific types of magnetic encoder ring defects, but only for the quality assessment. The article develops the quality assessment of magnetic encoder rings with the identification of encoder defects and the causes of their formation; hence, new parameters, not included in the standard parameters, are proposed: 4—σ, 5—$R_{+/-}$, 6—$C_{max}$; and 7—$D_{A}$. Together, all seven diagnostic parameters allow for unambiguous identification and determination of the cause of the three most common types of encoder ring degradation (Section 3.1, Section 3.2 and Section 3.3). The first three parameters were determined in the circumferential direction of the encoder, parameters 4—σ and 5—$R_{+/-}$ were determined in the radial direction of the magnetic encoder, and parameters 6—$C_{max}$ and 7—$D_{A}$ were determined in the two-dimensional plane, that is, the magnetic field distribution on the surface encoder. Below (Figure 11) is a short description in the form of a diagram of the listed diagnostic parameters divided into those included in the standards and those proposed by the authors of the article. Methods of determination and detailed analytical descriptions of the parameters are presented in the subchapters concerning particular types of degradation (Section 3.1, Section 3.2 and Section 3.3) for which the specific parameters were developed.

The reference values of the parameters included in the standards [10,11], $\left|B_{min}\right|$, Ndp and Dw were confirmed experimentally on the tested encoders (Figure 4) using the authors’ measuring stand (Section 2.1). The identification of magnetic encoder defects and the causes of their occurrence was based on experimentally determined reference values of the diagnostic parameters (Table 2) as a result of tests conducted on undamaged encoders.

### 4.1. Mechanical Degradation

Due to the mechanical degradation process, the absolute peak value of magnetic induction of the domains $\left|B_{min}\right|$ changed its value, and a variation in the magnetic induction values around the extreme changes was described by a statistical parameter, namely, the standard deviation σ of a discrete data series [17].

The decrease in the $\left|B_{min}\right|$ value is tantamount to the decrease in the magnetic induction value, as observed in the experiment (Section 3.1). A decrease in the variability around the ring’s magnetic induction radial extremum visually manifested itself as a flattening of the domain’s magnetic induction peaks in the magnetic field distribution (Figure 5a).

**Figure 12:**
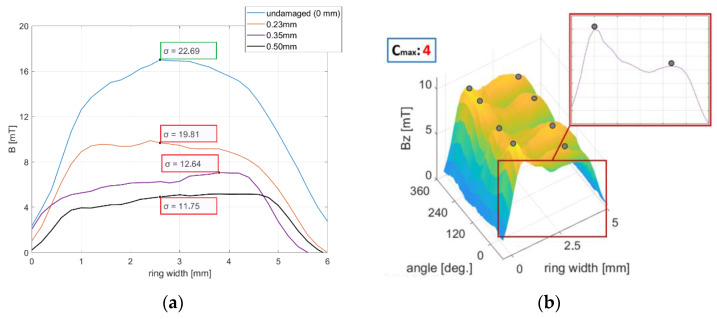
(**a**) Magnetic induction waveforms in the radial direction of mechanically degraded encoder rings. The standard deviation parameter σ was determined for each of the waveforms. (**b**) Envelope of the magnetic field induction distribution of the sample subjected to mechanical abrasion with cavities. For the envelope, the parameter C_max_ = 4 was calculated.

Figure 12a shows the waveforms of magnetic induction in the radial direction for samples subjected to four different mechanical degradation intensities, that is, the depths of magnetic layer abrasion (Table 1 For each of the four waveforms, the σ parameter was determined. With increasing damage intensity in the encoder ring’s radial direction, the parameter σ (Figure 12a) decreased in relation to its reference value for an undamaged encoder ring—σ=22.69 (Table 2).

For the purpose of irregular, specific types of mechanical degradation, abrasion with cavities (Figure 12b), the parameter $C_{max}$ was developed, which is related to the number of material cavities along the magnetic encoder ring circumference.

From the analytical description’s point of view, $C_{max}$ is equal to the number of sets of local maxima on the magnetic induction envelope of the encoder ring, Equation (9). A single set includes two or more local maxima max(B). These are local maxima with a given, minimal peak prominence [18] in the radial direction and a given angular length in the encoder ring circumferential direction.

(9) $$C_{max}=\overset{m}{\underset{j=1}{\cup }}{\left(max_{i}\left(B\right),max_{i+1}\left(B\right),\ldots max_{n}\left(B\right)\right)}_{m},\;n\geq 2$$

where $max_{i}\left(B\right)$ is the local maximum of the magnetic field induction envelope of the encoder ring, m is the number of local maxima sets, and n is the number of local maxima.

Figure 12b shows the envelope of the magnetic induction distribution of the encoder ring, which was subjected to mechanical abrasion at a 0.30 mm depth (Table 2). For the envelope (Figure 12b), the parameter $C_{max}=4$ was determined, which means that the tested sample had four cavities in the rubber–ferritic material layer.

To identify the mechanical degradation and to determine its intensity, the following parameters were used: the minimum; absolute peak value of magnetic induction of the domains $\left|B_{min}\right|$; the radial waveform’s standard deviation σ; and the maxima $C_{max}$ in the envelope of magnetic induction of the domains.

With increasing intensity of regular mechanical degradation, that is, depth of abrasion of the magnetic layer, parameters $\left|B_{min}\right|$ and σ decrease with respect to the required reference values: $\left|B_{min}\right|=16\;mT$ and σ=22,69. In the case of mechanical degradation with cavities, the $C_{max}$ parameter increased with the growth of cavities in the material. The reference value of the parameter was $C_{max}=0$ (Table 2) for no cavities.

### 4.2. Thermal Degradation

Stages I and II of thermal degradation proceeded, as described in the diagram in Figure 2.

**Figure 13:**
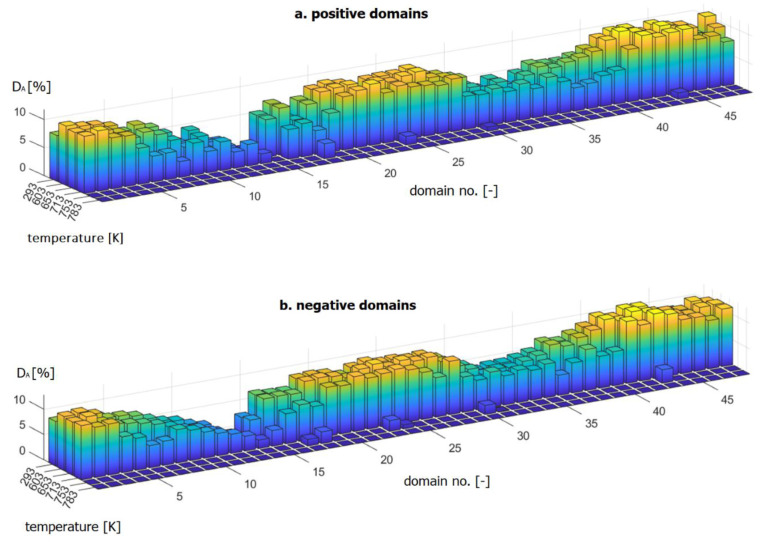
D_A_ areas of positive domains (**a**) and negative domain D_A_ areas (**b**) are shown as bar graphs.

At stage I of damage, at a destructive temperature of 653 K, there was a decrease in the minimum absolute value of magnetic induction of domains $\left|B_{min}\right|$ in relation to its reference values (Table 2), which is tantamount to a decrease in the magnetic induction value, as observed in the experiment (Section 3.2) and shown in Figure 13a.

In the next stage of thermal degradation of the encoder rings, for the measurement at the destructive temperature of 753 K, relative to the reference values (Table 2), there was a significant (in the range of 5 mT to 10 mT) (Figure 13b) decrease in $\left|B_{min}\right|$ and reduction in most of the positive and negative domain fields $D_{A}$ to 0% and several $D_{A}$ values to 0.5–1% (Figure 13).

$D_{A}$ allows for measurements of the area of a single domain above the determined magnetic induction threshold. The $D_{A}$ parameter is expressed as a percentage ratio of the domain area above the threshold $D_{A-THR}$ in relation to but not including the threshold domain area $D_{A-0}$ (10).

(10) $$D_{A}=\frac{D_{A-THR}}{D_{A-0}}\cdot 100\%$$

where $D_{A-THR}$ is the area of a single domain above the determined threshold of magnetic induction—140 mT and $D_{A-0}$ is the area of a single domain not including the threshold.

Figure 13 shows that the areas $D_{A}$ of both positive and negative domains did not change their size until a temperature of 713 K was reached—a transition temperature between the I and II stages of thermal degradation (Figure 7). However, for stage II of thermal degradation (at 753 K), most of the $D_{A}$ domain areas were reduced to 0%, and for a temperature of 783 K, all the $D_{A}$ areas of the domains did not exceed the value of 0.

In summary, the following parameters are required to identify thermal degradation and to determine their intensity: $\left|B_{min}\right|$ and $D_{A}$.

At stage I of thermal degradation (for destructive temperatures of 653 K and 713 K), there is a decrease in the domain magnetic induction peak values $\left|B_{min}\right|$ in relation to the reference value (Table 2).

At stage II of thermal degradation (for destructive temperatures: 753 K and 783 K), in addition to the aforementioned $\left|B_{min}\right|$ decrease, there is a decrease in the measured $D_{A}$ domain area values in relation to its reference value (Table 2).

### 4.3. Magnetic Degradation

In stage I of magnetic degradation, the domain magnetic induction minimum absolute peak value $\left|B_{min}\right|$ decreased. Parameter $\left|B_{min}\right|$ decreased due to an increase in the intensity of magnetic degradation, that is, the value of the destructive magnetic field intensity.

**Figure 14:**
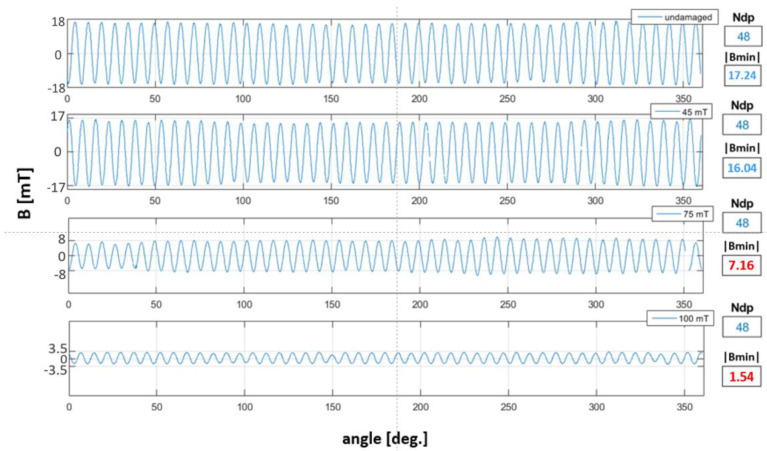
Circumferential magnetic induction waveforms of the reference encoder ring and samples at stage I of magnetic degradation. In the legends of each of the graphs, the values of the destructive field intensity are given. The Ndp and $\left|B_{min}\right|$ parameters for each waveform were determined.

Figure 14 shows the magnetic induction circumferential waveforms of the undamaged reference encoder ring and samples at stage I of magnetic degradation. The three encoders were subjected to increasing intensities of magnetic degradation. For each waveform (Figure 14), the parameters Ndp and $\left|B_{min}\right|$ were determined. The $\left|B_{min}\right|$ parameter, and accordingly, values of magnetic field induction decreased with increasing intensity of magnetic degradation. The results of Ndp and $\left|B_{min}\right|$ parameters are marked in red, which do not meet their reference values (Table 2).

**Figure 15:**
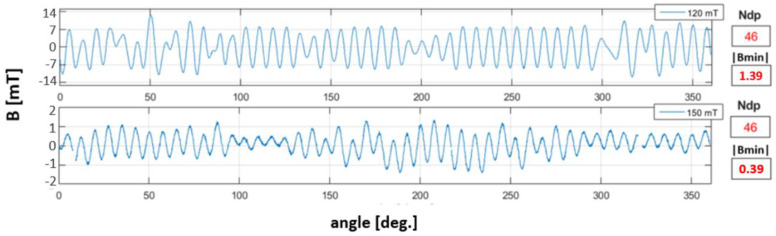
Circumferential waveforms of the magnetic induction signals of the encoder ring at stage II of magnetic degradation. In the legends of each of the graphs, the values of the destructive field intensity are given. The Ndp and $\left|B_{min}\right|$ parameters for each waveform were determined.

Figure 15 shows the circumferential magnetic induction waveforms of the encoder rings at stage II of magnetic degradation. Similarly, the waveforms at stage I of magnetic degradation (Figure 14), for stage II measurements, parameters $\left|B_{min}\right|$ and Ndp were determined. At stage II of degradation, in addition to the decrease in the $\left|B_{min}\right|$ value, there was a decrease in the encoder ring’s Ndp number. However, in contrast to the stage I of magnetic degradation (Figure 14), the reduction in the magnetic induction value was irregular along the circumference, which can be seen in the ring’s magnetic field induction on the surface distributions (Figure 10).

**Figure 16:**
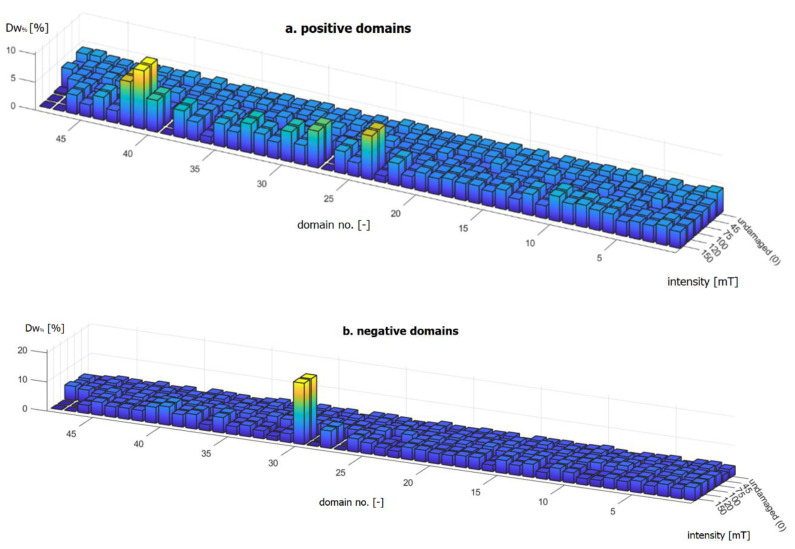
Positive domain widths (**a**) and negative domain widths (**b**) shown in bar graphs.

Figure 16 shows bar graphs of the domain widths Dw of the undamaged encoder ring and samples subjected to all five magnetic degradation intensities (Table 1). The graphs of the measured domain widths are expressed in percentage domain widths $Dw_{\%}$ above the required reference domain width $Dw_{ref}=1.64\;mm$ (Table 2). The mathematical notation of $Dw_{\%}$ is presented below as Equation (12):

(11) $$Dw_{\%}=\frac{Dw-Dw_{ref}}{Dw_{ref}}\cdot 100\%$$

where $Dw_{\%}$ is the measured domain width above the reference width, expressed in % Dw is the measured domain width, expressed in mm; and $Dw_{ref}$ is the reference domain width, expressed in mm.

Figure 16a,b represents domain widths that exceed the required reference value $Dw_{ref}$; on the OZ axis of the graph, the domain width was at a zero level. Domain widths that did not exceed $Dw_{ref}$ are not shown in the form of three-dimensional bars; instead, they are represented as flat rectangles (Figure 16).

Differences in the widths of both positive (Figure 16a) and negative (Figure 16b) domains occurred for the two highest damage intensities, namely, 120 mT and 150 mT, for the II magnetic degradation stage. Changes in domain widths in relation to the reference value (Table 2) were caused by a decrease in the domain pair number Ndp (Figure 16). For negative domains (Figure 16b), the largest measured domain widths at stage II of magnetic degradation reached 20% above $Dw_{ref}$. However, the positive domain widths (Figure 16a) in stage II of magnetic degradation were 5% to 10% greater than the $Dw_{ref}$ value. Correspondingly, some of the domain widths on the encoder ring circumference decreased. For 120 mT and 150 mT magnetic degradation intensities, there were domains that did not exceed $Dw_{ref}$, represented in the form of flat rectangles in the graphs (Figure 16).

The characteristic of stage II of magnetic degradation was the change in domain sign in the radial direction of the magnetic encoder ring (Figure 10). Therefore, a parameter $R_{+/-}$ was developed, which allows for detecting changes in the sign of the magnetic induction vector within a single domain. It is a two-state logical parameter YES/NO, where YES means a change in sign, and NO means no sign change within the domain. Similar to the domain width parameter Dw (9), to determine $R_{+/-}$, the sgn(B) function (6) for the magnetic induction vector was calculated. Then, using the forward difference quotient method (12), the discrete derivative of sgn(B) was calculated. Hence, the derivative of f(x) at x=a is as follows:

(12) $${\left[\frac{df}{dx}\right]}_{x=x_{i}}=\underset{x\to a}{lim}\frac{f\left(x_{i+1}\right)-f\left(x_{i}\right)}{x_{i+1}-x_{i}},\;for\;a=\left\{-1,1\right\}$$

where $x_{i}$ is the consecutive value of the magnetic field induction vector and a is the set of sgn(B) vector values.

The derivative ${\left[\frac{df}{dx}\right]}_{x=x_{i}}$, depending on the value at point a, takes zero values or values different than zero. Hence, the parameter $R_{+/-},$ Equation (13), assumes the YES or NO state for the derivative values of Equation (12) equal to zero or different than zero:

(13) $$R_{+/-}=\{\begin{array}{c} {\left[\frac{df}{dx}\right]}_{x=x_{i}}\neq 0\to YES\\ {\left[\frac{df}{dx}\right]}_{x=x_{i}}=0\to NO \end{array}$$

where $R_{+/-}$ is the single domain sign change detection parameter, YES is the logical state for change in sign, and NO is the logical state for no sign change.

**Figure 17:**
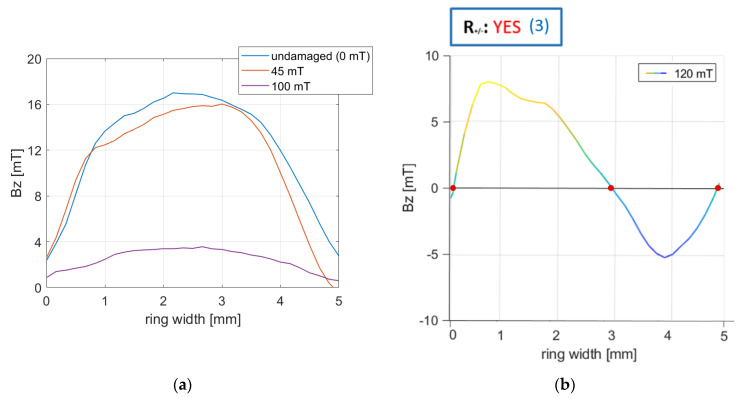
(**a**) Radial waveforms of encoder rings subjected to two intensities of magnetic degradation at stage I compared to the undamaged original encoder ring. (**b**) Radial waveform of the encoder ring at stage II of magnetic degradation, 120 mT intensity. The parameter R_+/−_ sign change was determined for the waveform. The red marker marks the zero crossing points of the magnetic induction.

Figure 17a shows encoder rings subjected to two intensities of magnetic degradation at stage I compared to the undamaged original encoder ring. At stage I of magnetic degradation, within a single domain, no sign changes were observed (all magnetic induction values were of the same sign). Therefore, for each waveform (Figure 17a), parameter $R_{+/-}=NO$. Figure 17b shows the ring’s magnetic induction radial waveform on stage II of the degradation, with 120 mT intensity. For the waveform (Figure 17b), the parameter $R_{+/-}$ was calculated. Using the $R_{+/-}$ parameter, it was possible to detect sign changes and their number. $R_{+/-}=YES\left(3\right)$ means that within one domain, there were three magnetic induction sign changes.

The identification and determination of the intensity of magnetic degradation at its two stages is possible due to the magnetic field parameters of the encoder ring: Ndp, Dw; and $R_{+/-}$.

In stage I of degradation, along with the increase in the intensity of magnetic degradation, i.e., the intensity of the destructive field, the minimum absolute peak value of the magnetic induction $\left|B_{min}\right|$ (Figure 14) relative to its reference value (Table 2) decreased. In stage II of magnetic degradation, along with the increase in the destructive field strength, in addition to the aforementioned decrease in the $\left|B_{min}\right|$ value, the number of domain pairs Ndp decreased in relation to the reference value Ndp=48. Thus, the reference width of the Dw domains was not met (Table 2), and there was a change in sign within a single domain, which resulted in a state change in the $R_{+/-}$ parameter.

## 5. Discussion

Based on the review of many standard requirements of encoder recipients, common parameters for the evaluation of the encoder quality for all analyzed standards were determined, and proprietary parameters were proposed. The results (Section 3.1–3.3) showed that with the use of the proposed diagnostic parameters, i.e., 1—$\left|B_{min}\right|$, 2—Ndp, 3—Dw, 4—$D_{A}$, 5—σ, 6—$R_{+/-}$ and 7—$C_{max}$, it was possible to identify encoder defects and evaluate the defect degradation progress.

Each examined ring damaging phenomenon has its own specific nature; therefore, an individual approach is required. Nevertheless, in the case of mechanical degradation and the magnetic field, it was possible to find parameters that indicate the early stages of damage development. In the case of thermal degradation, it was not so simple. Despite the decrease in the $\left|B_{min}\right|$ value in stage I of thermal degradation (Section 3.2), the magnetic encoder ring still met the reference value of this parameter (Table 2); that is, the decrease in the value $\left|B_{min}\right|$ was too small. Therefore, the ring did not lose its encoder functionality. In addition, degradation by the magnetic field turned out to be the most complex because depending on the degree of development, we could distinguish quantitative and then qualitative changes. To summarize, the variability or constancy of specific parameters identifies specific types of degradation and describes the stages of their development. Table 3 includes a set of parameters and their capability description referring to three selected types of coder degradation. It was not possible to define one parameter that would sufficiently describe each type of degradation, but it is possible choose the right set of parameters depending on what is needed. The green frame indicates degradation, for which the parametric diagnostic result still meets the quality in relation to the parameters reference values contained in Table 2. At stage I of thermal degradation, despite the loss of some of the magnetic properties (Section 3.2), the encoder will perform the functionality of measuring rotational motion parameters.

To date, it has been difficult to find any measurement method to ensure the identification of the causes of encoder defects, which makes the proposed measurement method promising. It is difficult to differentiate such reasons with full certainty because many scenarios can degrade the encoder and sometimes there are few causes simultaneously. That is why the research should proceed. One study shows that it is important to ensure the right combination of measurement technology adapted to the geometry, dimensions and specific research problems, such as the stage of degradation or reason for degradation of magnetic encoders. Additionally, referring to the proposed parameters, it is possible to perform an encoder quality assessment that will address the requirements contained in the recent standards of encoder recipients. The parametric form of the measurement result makes it possible to use it in industry where the quality assessment process must be automated.

In summary, the presented possibilities for evaluating encoder ring degradation can be used to diagnose systems recording motion parameters. This is extremely important from the perspective of industrial practice. In addition, confirmation of the reliability of the indications of such a system can be the basis for a modern approach, that is, diagnosing the technical condition of rotating machines based on the magnetic signal from the encoder.

## Figures and Tables

**Table 1:** Types and intensities of magnetic encoder ring degradations.

	Type of Degradation			
	No.	A. Mechanical Abrasion [mm] *	B. Magnetic Field [mT] **	C. Temperature [K]
**INTENSITY**	**1.**	0.00	0	293 ***
	**2.**	0.23	45	603
	**3.**	0.25	75	653
	**4.**	0.30 (+ cavities)	100	713
	**5.**	0.35	120	753
	**6.**	0.50	50	783

*—max. depth of magnetic layer—0.70 mm, **—encoder ring nominal magnetic field—10 mT, *** ambient temperature.

**Table 2:** Reference values of the diagnostic parameters.

**Parameter**	$|B_{min}|\geq 16\;\mathrm{mT}$
	Ndp=48
	$D_{w}=1.64\;\mathrm{mm}$
	$D_{A}>0$
	σ≥22.69
	$R_{+/-}=NO$
	$C_{max}=0$

**Table 3:** List of parameters for particular types of degradation. The green frame shows the type of degradation for which the parametric diagnostics result indicates meeting the quality of the encoder ring.

		A. Magnetic Field		B. Machnical Abrasion		C. Heat	
		I Stage	II Stage	Regular	+Cavities	I Stage	II Stage
**PARAMETERS**	**1.** $\;\left|B_{min}\right|$	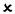	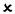	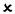	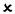	**✓**	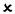
	**2.** Ndp	**✓**	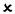	**✓**	**✓**	**✓**	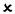
	**3.** Dw	**✓**	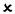	**✓**	**✓**	**✓**	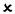
	**4.** $D_{A}$	**✓**	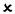	**✓**	**✓**	**✓**	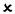
	**5.** σ	**✓**	–	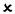	**–**	**✓**	–
	**6.** $R_{+/-}$	**✓**	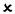	**✓**	**✓**	**✓**	**✓**
	**7.** $C_{max}$	–	–	**✓**	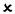	–	–

Legend: **✓** correct; 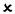 wrong; **—** not applicable.

## Data Availability

Not applicable.
